# Editorial: Brief interventions in suicide prevention across the continuum of care

**DOI:** 10.3389/fpsyt.2022.976855

**Published:** 2022-07-26

**Authors:** Peter C. Britton, Sofian Berrouiguet, Natalie B. Riblet, Bao-Liang Zhong

**Affiliations:** ^1^Veterans Integrated Services Network (VISN) 2 Center of Excellence for Suicide Prevention, Department of Veteran Affairs, Finger Lakes Healthcare System, Canandaigua, NY, United States; ^2^Department of Psychiatry, University of Rochester Medical School, Rochester, NY, United States; ^3^Université Bretagne Occidentale, Brest, France; ^4^Veterans Affairs Medical Center, White River Junction, VT, United States; ^5^Department of Psychiatry, Geisel School of Medicine at Dartmouth College, Hanover, NH, United States; ^6^Department of Psychiatry, Wuhan Mental Health Center, Wuhan, China; ^7^Affiliated Wuhan Mental Health Center, Tongji Medical College of Huazhong University of Science and Technology, Wuhan, China; ^8^Research Center for Psychological and Health Sciences, China University of Geosciences, Wuhan, China

**Keywords:** suicide, prevention, intervention, treatment, international

Suicide prevention has become a global priority (1), inspiring exciting developments in suicide prevention research (2), and a growing interest in brief interventions (3). In our request for papers for this special edition, we broadly defined brief interventions to include treatments that are straightforward, have minimal face-to-face contact, and/or are of brief duration, and that address outcomes that are associated with suicidal behavior including treatment engagement, psychological and physical problems related to suicide, means-safety, suicidal ideation, and non-fatal and fatal attempts themselves. In this introduction to the special edition, we explore brief suicide intervention research from public health, clinical research, and mechanistic perspectives. We then examine the papers in this special edition to provide examples of what can be learned from brief intervention research and highlight opportunities to advance the field. Finally, we consider their implications for suicide prevention efforts globally, to increase their scientific and preventive potential.

## Effectiveness of brief interventions

Despite their diminutive nature, structured reviews and meta-analyses of brief suicide interventions suggest that these interventions have considerable potential to improve suicide-related outcomes. In a systematic review of two-way interventions of up to three treatment sessions with follow-up care, McCabe et al. (4) identified four trials, with one trial showing a significant reduction in suicide deaths, two showing reductions in risk for non-fatal attempts, and one showing reduced depression. Similarly, in a systematic review and meta-analysis of brief interventions initiated in emergency departments, Inagaki et al. (5) found support for active contact and follow-up in reducing risk for non-fatal attempts. In another recent meta-analyses of single session interventions with follow-up care, Doupnik et al. (6) determined that brief interventions not only reduced the risk for non-fatal attempts but also increased linkage to follow-up care. However, they found no impact of brief suicide interventions on depressive symptoms. Brief intervention research has also been informed by technological developments. In a systematic review and meta-analysis of self-guided digital interventions, Stefanopoulou et al. (7) concluded that there is not enough information to determine whether self-guided digital intervention reduce suicidal ideation and behavior. However, Torok et al. (8) determined that self-guided digital interventions reduced suicidal ideation, with the reduction being significant for interventions that focus on suicide but not for those that focus on psychiatric symptoms. Thus, brief interventions have promising potential to impact suicide prevention efforts, with important implications for healthcare systems and suicide prevention research. It is also important to note that the two clinical interventions that have been found to reduce risk for suicide deaths, caring contacts (9), and the World Health Organization (WHO) Brief Intervention and Contact (BIC) (10), can be conceptualized as brief interventions.

## Public health perspective

Rose's Theorem enabled researchers to recognize “*that a large number of people at small risk may give rise to more cases of disease than a small number who are at high risk* (11).” It follows that reducing suicide risk in larger populations increases the potential for reducing suicide rates (12, 13), which also has implications for reducing suicide *within* high-risk populations. A straightforward and brief intervention that can be disseminated across a large number of high-risk individuals may have greater preventive potential than a complex and lengthy intervention that can only be disseminated across a small number of high-risk individuals. The WHO research team randomized 1,867 participants from five countries to receive either BIC, which consisted of education with follow-up or treatment as usual and found there were significantly fewer deaths in the BIC group than in the treatment as usual condition over 18 months (10). The intervention was straightforward and brief enough to be studied across five different sites internationally, with an outcome of suicide deaths, illustrating the international potential of brief interventions. To date, no other clinical intervention has been tested on a global scale with suicide as the primary outcome (14).

## Clinical research perspective

In 1967, Gordon Paul noted that the goal of intervention research is to identify “What treatment, by whom, is most effective for this individual with that specific problem, and under which set of circumstances (15)?” However, an incredible number of well-designed and conducted studies are needed to answer these questions. Because brief intervention studies are easier to conduct than more complex interventions, it is becoming possible to answer these questions. In the original caring contacts study, Motto and Bostrom randomized 845 patients who had been psychiatrically hospitalized for depression or suicide risk and refused treatment to receive a series of caring contacts or no contacts. The researchers found that the caring contacts group had significantly lower rates of suicide over the first 24 months of follow-up when compared to the control condition (9). A recent meta-analysis identified an additional five studies of caring contacts that were conducted over the two decades that followed and found that caring contacts did not reduce risk for suicide overall (16). Nevertheless, at 2-year follow-up, two of the studies found a reduction in risk, whereas one found an increase in risk. If caring contacts reduces risk for suicide, it is likely that it is most effective in subpopulations such as the original group of hospitalized depressed and suicidal patients who refused additional treatment. However, identifying the populations that stand to benefit the most from suicide prevention interventions becomes extremely challenging if not impossible when considering complex interventions that have not yet been tested with suicide as an outcome.

## Mechanism research perspective

Knowing that an intervention works and who it works for often tells us little about why it works, which is the domain of mechanism research. Mechanisms of change explain “why treatment works, through what processes, and how change comes about (17).” Brief interventions lend themselves to investigation of treatment mechanisms because their target mechanisms must be narrow in scope. Outcomes of studies using brief interventions therefore tell us something about the mechanisms whereby change is achieved, even if the mechanism was not measured. Both BIC and caring contacts, for example, make effort to maintain contact with at-risk individuals with positive results. From this perspective, studies of brief interventions can also be conceptualized as akin to component analyses (17). Rather than conducting dismantling studies of complex interventions to identify essential components, researchers are studying the components themselves. Combining the results from multiple brief intervention studies can help us answer important questions, such as: Is there a group of suicidal individuals that is responsive to any effective brief intervention? Are different brief interventions needed for different at-risk groups? Are more brief interventions that target different mechanisms better than targeting a single mechanism? Considering BIC and caring contacts, meta-analyses suggest that using caring contacts may have no or only a small effect (9). However, would using both BIC and caring contacts improve outcomes? Or would it be better to provide BIC to patients who accept treatment and caring contacts to those who do not?

## Lessons learned from included papers

The studies that are included in this special edition provide additional examples of how brief intervention research is being used to learn more about suicide prevention efforts. Policy work that focuses on reducing access to potentially lethal means such as firearms has been shown to reduce risk for suicide (18–20). When there is little hope of changes in firearm policy, clinicians are forced to address firearm safety in-session. Asarnow et al. tested the acceptability of a web-based decision aid to promote means safety among parents of children in crisis. By testing a brief web-based interface, this study examines the potential use of technology to facilitate potentially difficult conversations regarding firearm access and safety. The public health potential of such interventions is considerable as the digital interface may make it easier to broach this difficult topic on a broad scale (21). The inclusion of user-centered design to develop the decision aid also highlights the importance of involving both patients and families in the development of suicide prevention interventions.

Brief interventions such as caring contacts can be tailored to meet the needs of patients that are treated within specific healthcare systems. Landes et al. described a project to implement caring contacts across Department of Veterans Affairs (VA) emergency departments. Implementation across a healthcare system, even of an intervention as straightforward as sending a caring letter, is an enormous endeavor. Such efforts require intimate knowledge of healthcare systems, which requires the input of multiple groups of people at multiple levels within the organization. Landes et al. describe the process they undertook to gather such information, to tailor the caring contacts intervention to VA emergency departments, and to do so in a way that is welcomed by both patients and providers. In doing so, they are creating a template that other healthcare systems and research can follow when considering the implementation of caring contacts.

The papers also contribute to our scientific knowledge of how individual's responses during brief treatments can impact the intervention itself. Safety planning interventions were developed to provide high-risk individuals with tools that would enable them to resist suicidal urges for brief periods of time (22), and there is growing support for their efficacy and effectiveness (23–25). In a small study of warning signs among individuals who received Crisis Response Planning (CRP), a safety planning intervention, Bauder et al. did not find systematic differences in warning signs across important demographic categories and gun ownership. They did, however, confirm that males completing a CRP were more likely to own a firearm than females, reminding us of the need to address firearm means safety in safety planning interventions with males. To our knowledge, means safety outcomes have never been studied in the context of safety planning, highlighting a potential step forward regarding the potential impact of safety plans.

These papers also contributed to our understanding of the mechanisms whereby brief interventions may work. In a randomized controlled trial of 120 patients who recently attempt suicide, the Attempted Suicide Short Intervention Program (ASSIP) program was found to reduce risk for non-fatal suicide attempts by 80% over 2 years when compared to care as usual (26). In this mechanism study, Gysin-Maillart et al. found that reasons for dying fell for both the ASSIP and control group over follow-up, but that the reduction was larger in the ASSIP condition. In contrast, they found no overall effect on reasons for living. This suggests that ASSIP may have been effective because it reduced reasons for dying more than treatment as usual. Multiple interpretations are plausible, including the authors own interpretation that effective suicide prevention interventions target reasons for dying. However, another possibility is that ASSIP did not change reasons for living because it did not target them, and that there may be untapped potential in adding a component that does.

## Potential implications for global suicide prevention efforts

Suicide prevention is an international problem. Brief suicide prevention interventions are among the few suicide prevention efforts that have shown success in reducing risk for suicide. Their brevity makes them easier to study with large samples, with potential to study their impact on suicide deaths. When they are found to be efficacious, they are also easier to implement across healthcare systems as they require fewer resources than more complex interventions, which increases the potential of brief interventions to prevent suicide deaths. Finally, because they are of limited scope and/or duration, their effectiveness or ineffectiveness can be used to learn about potential mechanisms of action, knowledge that can be used to inform future research. If suicide prevention interventions are going to play a role in reducing suicides globally, utilization and research on brief interventions may require further consideration.

## Author contributions

PB wrote the first draft. SB, NR, and B-LZ revised the manuscript. All authors have read and approved the submitted version.

## Funding

NR has support from the Department of Veterans Affairs Clinical Science Research and Development Career Development Award Program (MHBC-007-19F).

## Conflict of interest

The authors declare that the research was conducted in the absence of any commercial or financial relationships that could be construed as a potential conflict of interest.

## Publisher's note

All claims expressed in this article are solely those of the authors and do not necessarily represent those of their affiliated organizations, or those of the publisher, the editors and the reviewers. Any product that may be evaluated in this article, or claim that may be made by its manufacturer, is not guaranteed or endorsed by the publisher.

## References

[1] World Health Organization. Preventing Suicide: A Global Imperative. Geneva: World Health Organization (2016).

[2] Mann JJ Michel CA Auerbach RP. Improving suicide prevention through evidence-based strategies: a systematic review. Am J Psychiatry. (2021) 178:611–24. 10.1176/appi.ajp.2020.20060864 33596680 PMC9092896

[3] Knipe D Padmanathan P Newton-Howes G Chan LF Kapur N. Suicide and self-harm. Lancet. (2022) 399:1903–16. 10.1016/S0140-6736(22)00173-8 35512727

[4] McCabe R Garside R Backhouse A Xanthopoulou P. Effectiveness of brief psychological interventions for suicidal presentations: a systematic review. BMC Psychiatry. (2018) 18:1–13. 10.1186/s12888-018-1663-5 29724203 PMC5934886

[5] Inagaki M Kawashima Y Yonemoto N Yamada M. Active contact and follow-up interventions to prevent repeat suicide attempts during high-risk periods among patients admitted to emergency departments for suicidal behavior: a systematic review and meta-analysis. BMC Psychiatry. (2019) 19:1–11. 10.1186/s12888-019-2017-7 30683075 PMC6347824

[6] Doupnik SK Rudd B Schmutte T Worsley D Bowden CF McCarthy E. Association of suicide prevention interventions with subsequent suicide attempts, linkage to follow-up care, and depression symptoms for acute care settings: a systematic review and meta-analysis. JAMA psychiatry. (2020) 77:1021–30. 10.1001/jamapsychiatry.2020.1586 32584936 PMC7301305

[7] Stefanopoulou E Hogarth H Taylor M Russell-Haines K Lewis D Larkin J. Are digital interventions effective in reducing suicidal ideation and self-harm? A systematic review. J Ment Health. (2020) 29:207–16. 10.1080/09638237.2020.1714009 31989852

[8] Torok M Han J Baker S Werner-Seidler A Wong I Larsen ME. Suicide prevention using self-guided digital interventions: a systematic review and meta-analysis of randomised controlled trials. Lancet Digit Health. (2020) 2:e25–36. 10.1016/S2589-7500(19)30199-2 33328037

[9] Motto JA Bostrom AG. A randomized controlled trial of postcrisis suicide prevention. Psychiatr Serv. (2001) 52:828–33. 10.1176/appi.ps.52.6.828 11376235

[10] Fleischmann A Bertolote JM Wasserman D De Leo D Bolhari J Botega NJ. Effectiveness of brief intervention and contact for suicide attempters: a randomized controlled trial in five countries. Bull World Health Organ. (2008) 86:703–9. 10.2471/BLT.07.046995 18797646 PMC2649494

[11] Rose G. Sick individuals and sick populations. Int J Epidemiol. (1985) 14:32–8. 10.1093/ije/30.3.427 3872850

[12] Sher L. Is it time to employ Rose's Theorem to prevent suicide? Aust NZ J Psychiatry. (2019) 53:381. 10.1177/0004867419829220 30760001

[13] Yip PSF So BK Kawachi I Zhang Y. A Markov chain model for studying suicide dynamics: an illustration of the Rose theorem. BMC Public Health. (2014) 14:1–6. 10.1186/1471-2458-14-625 24948330 PMC4082176

[14] Riblet NBV Shiner B Young-Xu Y Watts BV. Strategies to prevent death by suicide: meta-analysis of randomised controlled trials. Br J Psychiatry. (2017) 210:396–402. 10.1192/bjp.bp.116.187799 28428338

[15] Paul GL. Strategy of outcome research in psychotherapy. J Consult Psychol. (1967) 31:109. 10.1037/h0024436 5342732

[16] Skopp NA Smolenski DJ Bush NE Beech EH Workman DE Edwards-Stewart A. Caring contacts for suicide prevention: a systematic review and meta-analysis. Psychol Serv. (2022). 10.1037/ser0000645 35420858

[17] Kazdin AE. Mediators and mechanisms of change in psychotherapy research. Annu Rev Clin Psychol. (2007) 3:1–27. 10.1146/annurev.clinpsy.3.022806.091432 17716046

[18] Anestis MD Anestis JC. Suicide rates and state laws regulating access and exposure to handguns. Am J Public Health. (2015) 105:2049–58. 10.2105/AJPH.2015.302753 26270305 PMC4566524

[19] Chapman S Alpers P Agho K Jones M. Australia's 1996 gun law reforms: faster falls in firearm deaths, firearm suicides, and a decade without mass shootings. Inj Prev. (2006) 12:365–72. 10.1136/ip.2006.013714 17170183 PMC2704353

[20] Kapusta ND Etzersdorfer E Krall C Sonneck G. Firearm legislation reform in the European Union: impact on firearm availability, firearm suicide and homicide rates in Austria. Br J Psychiatry. (2007) 191:253–7. 17766767 10.1192/bjp.bp.106.032862

[21] Murray E Hekler EB Andersson G Collins LM Doherty A Hollis C. Evaluating digital health interventions: key questions and approaches. Am J Prev Med. (2016) 51:843–51. 10.1016/j.amepre.2016.06.008 27745684 PMC5324832

[22] Stanley B Brown GK. Safety planning intervention: a brief intervention to mitigate suicide risk. Cogn Behav Pract. (2012) 19:256–64. 10.1016/j.cbpra.2011.01.00131112330

[23] Bryan CJ Mintz J Clemans TA Leeson B Burch TS Williams SR. Effect of crisis response planning vs. contracts for safety on suicide risk in U.S. Army soldiers: a randomized clinical trial. J Affect Disord. (2017) 212:64–72. 10.1016/j.jad.2017.01.028 28142085

[24] Stanley B Brown GK Brenner LA Galfalvy HC Currier GW Knox KL. Comparison of the safety planning intervention with follow-up vs usual care of suicidal patients treated in the emergency department. JAMA Psychiatry. (2018) 75:894–900. 10.1001/jamapsychiatry.2018.1776 29998307 PMC6142908

[25] Miller IW Camargo CA Jr Arias SA Sullivan AF Allen MH. Suicide prevention in an emergency department population: the ED-SAFE study. JAMA Psychiatry. (2017) 74:563–70. 10.1001/jamapsychiatry.2017.0678 28456130 PMC5539839

[26] Gysin-Maillart A Schwab S Soravia L Megert M Michel K. A novel brief therapy for patients who attempt suicide: a 24-months follow-up randomized controlled study of the attempted suicide short intervention program (ASSIP). PLoS Med. (2016) 13:e1001968. 10.1371/journal.pmed.1001968 26930055 PMC4773217

